# Formulation Strategies for Improving the Stability and Bioavailability of Vitamin D-Fortified Beverages: A Review

**DOI:** 10.3390/foods11060847

**Published:** 2022-03-16

**Authors:** Elsa F. Vieira, Suene Souza

**Affiliations:** 1 REQUIMTE/LAQV, Polytechnic of Porto—School of Engineering, Rua Dr. António Bernardino de Almeida, 4249-015 Porto, Portugal; 2 Faculty of Nutrition and Food Sciences, Rua do Campo Alegre, 4150-180 Porto, Portugal; suene.vs@gmail.com

**Keywords:** vitamin D-fortified beverages, delivery systems, stability, bioavailability

## Abstract

Vitamin D is a lipophilic bioactive that plays an important role in bone health. Fortification of beverages, such as milk, fruit juices, teas, and vegetable drinks, could be an efficient strategy to prevent vitamin D deficiency and its associated effects on health. This review summarizes the current understanding of beverage fortification strategies with vitamin D and the resulting effects on the stability, bioaccessibility, and sensory properties of the formulated products. The direct addition technique has been the conventional approach to fortifying beverages. In addition, encapsulation has been pointed out as a desirable delivery approach to increase stability, preserve bioactivity, and enhance the absorption of vitamin D in beverage systems. The literature reports the potential applicability of several methods for encapsulating vitamin D in beverages, including spray drying, micro/nanoemulsions, nanostructured lipid carriers, liposomes, and complexation to polymers. Some of these delivery systems have been assessed regarding vitamin D stability, but there is a lack of kinetic data that allow for the prediction of its stability under industrial processing conditions. Moreover, in some cases, the applicability of some of these delivery systems to real beverages as well as the in vivo efficacy were not evaluated; thus, fortification strategies with a global outreach are lacking.

## 1. Introduction

Vitamin D deficiency (VDD) is one of the most common micronutrient malnutrition disorders globally, affecting both developed and developing countries [1]. Its worldwide prevalence is up to 1 billion [2], a reason why some authors have pointed to VDD as a pandemic situation [3,4,5]. As exposure to UV rays is the main natural source of vitamin D3 (VD3), low sunlight exposure, due to factors such as seasonality, lifestyle, and use of sunscreens, greatly contribute to the prevalence of VDD [6,7,8]. Another contributing factor is vitamin D ingestion below the recommended dietary value (RDA), i.e., 10 µg per day for infants below 12 months, 15 µg per day for adults and 20 µg per day for the elderly above 70 years old [9]. Satisfying the RDA of vitamin D is a challenge given the limited availability of food sources of this nutrient [8].

**Figure 1:**
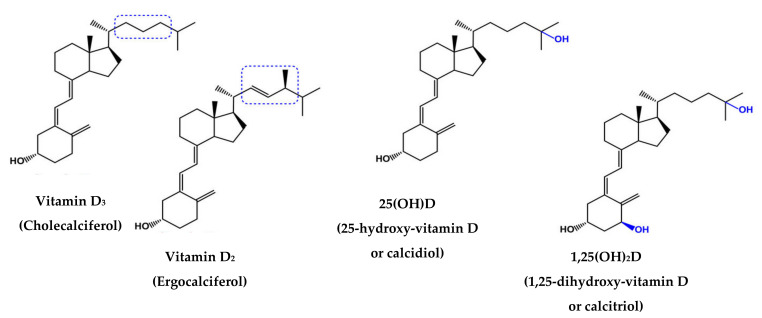
Chemical structures of vitamins D3, D2, 25(OH)D and 1,25(OH)_2_D. Structural differences are highlighted in blue. Adapted from [12].

Chemically, vitamin D is a lipophilic compound with two prevalent forms, ergocalciferol (vitamin D2, VD2), which is synthesized by ultraviolet radiation from plants, and cholecalciferol (VD3), which is formed in human skin and is present in animal foods [10]. Their chemical forms are illustrated in Figure 1; VD2 has a double bond between carbons 22 and 23 and a methyl group in its side chain, unlike VD3 [11].

The in vivo effectiveness of both vitamers is still not fully clarified, i.e., some studies showed the same biological activity while other studies suggested a higher bioavailability for VD3 [13]. Upon ingestion, both forms are converted into the same biologically active form, 1,25-dihydroxyvitamin D [1,25(OH)_2_D] [13]. The marker for vitamin D status is serum 25-hydroxyvitamin D [25(OH)D]; a value higher than 50 nmol/L or 20 ng/mL is correlated with disease prevention and regulation of physiological functions [14]. Vitamin D is essential for the regulation of multi-system functions, namely, phosphorus and calcium metabolism [15], muscle health and bone mineralization, insulin reactivity, cell differentiation and prevention of cardiovascular [16], cerebrovascular [17] and autoimmune diseases such as diabetes [18] and cancer [19]. VDD has been also associated with depression and increased risk of mortality [20]. In adults and children, the most common bone disorders related to VDD are rickets and osteomalacia [21].

The World Health Organization & Food and Agriculture Organization of the United Nations (WHO/FAO) have suggested different approaches to treat/prevent VDD, including increased intake of vitamin D dietary sources, vitamin D supplements and vitamin D-fortified foods and beverages [22]. Although the food fortification strategy is considered an excellent approach to prevent VDD [23,24], it has some technological challenges for food industry. Delivery of vitamin D in food/beverages should exhibit desirable physicochemical characteristics and guarantee bioaccessible and bioavailable vitamin D without affecting the sensory properties of the final product [25]. Like other lipophilic nutrients and organic compounds, vitamin D is an unstable molecule that can degrade when exposed to cooking, pasteurisation, sterilisation, drying, and in the presence of light and oxygen during the storage period [26,27]. These issues become even more challenging for vitamin D fortification of beverages due its high sensitivity to oxidation, hydrophobic characteristics, low aqueous solubility, and acid sensitivity [26,28]. To answer these limitations, several techniques, namely emulsions and encapsulation, have been adopted to improve the homogeneous distribution of vitamin D in the beverage matrix, as well as its stability and bioavailability by protecting against oxidative processes [26,29]. So far, several food products have been fortified with vitamin D, including milk and dairy products (e.g., cheese, butter, yoghurt), wheat flour, breakfast cereals and bread [8,26]. Although the effectiveness of some of these food vehicles for a vitamin D fortification strategy was recently reviewed by several authors [30,31,32], including milk and dairy products [30,33], to our best knowledge there is no review on the preparation of other kinds of vitamin D-fortified beverages, namely fruit juices, teas, and vegetable drinks. This prompted us to compile literature about the vitamin D fortification of all these types of beverages, including the information about the fortification methods applied and its effects on the stability, bioaccessibility, and sensory properties of the final products.

## 2. Examples of Vitamin D-Fortified Beverages

Several beverages have been fortified with vitamin D, such as milk, fruit juices, vegetable drinks and tea. Table 1 presents some examples of these beverages and summarizes the information regarding the fortification level applied and the main effects of processing (e.g., temperature, humidity, pH, and ionic strength) and storage conditions on the stability and bioaccessibility of vitamin D in the formulated products.

Milk has been widely used as a vehicle for vitamin D fortification because it is a staple food with good implementation and acceptance by the population [7,26]. Hanson and Metzger [34] observed that 2% fat milk and low-fat chocolate milk are good fortification vehicles of VD3. In both cases, the fortification level used was 100–250 international-units (IU)/240 mL and no losses of VD3 and changes in sensory characteristics of the formulated products were observed after heat treatment and a storage period of 21, 60 and 42 days. Accordingly, two studies conducted in India found good stability of VD2 in fortified milks [37,38]. The research conducted by Kaushik et al. [38] showed that VD2 used to fortify a mixture of cow and buffalo milk (600 IU/L) remained stable in processing, packaging, and storage conditions. Furthermore, Syama et al. [37] observed that complexation of VD2 to milk protein improves its stability during light exposure and heat treatment. Regarding the effect of storage conditions, authors observed that VD2–protein complexes showed the highest stability at −20 °C and lowest stability at 37 °C. To date, only two clinical studies observed positive effects on health from the ingestion of vitamin D-fortified milk [35,36]. The 12-week intervention conducted by Khadgawat et al. [36] showed an increase in serum 25(OH)D levels in Indian children (10–14 years) after ingestion of VD3-fortified-milk (600–1000 IU/200 mL) Similarly, Neyestani et al. [35] showed that the ingestion of VD3-fortified milk (100 IU/200 mL) for 12 weeks can increase the serum 25(OH)D levels of Iranian children (9–12 years old).

“Lassi”, a milk-based beverage of Indian origin, was also used for vitamin D fortification. The study conducted by Maurya and Aggarwal [39] showed that fortified “Lassi” with microencapsulated VD3 had a high stability and sensorial acceptability. Another milk-based beverage fortified with vitamin D was goat-milk kefir. Fauziyyah et al. [40] evaluated the effects of fermentation on VD3 fortified content, as well as on the sensory and physicochemical quality of goat-milk kefir. The authors recommended that the fortification of VD3 (42 IU/100 mL) can be carried out within 6 h of fermentation and suggested encapsulation of vitamin D to increase the VD3 stability of the product.

To address issues such as intolerance and allergy to milk and/or vegan-based diets, alternative vegetable beverages to milk have been considered [35]. Orange juice fortified with vitamin D has been widely used as a vitamin D fortification strategy in Finland, the USA, and Canada [7]. Table 1 gives four examples of clinical trials showing that orange juice fortified with vitamin D can increase serum 25(OH)D levels in the population [35,41,42,43]. Tangpricha et al. [41] observed that VD3-fortified orange juice (1000 IU/240 mL) increased the serum 25(OH)D concentrations of adults by >150% over a 12-week intervention. Biancuzzo et al. [42] observed that VD2 and VD3 fortification in orange juice (at the same level of 1000 IU/240 mL) are similarly effective at increasing the serum 25(OH)D concentrations in adults in an 11-week intervention. Economos et al. [43] found that ingestion (for 12 weeks) of VD3-fortified orange juice (100 IU/240 mL) increased the serum 25(OH)D levels of children with an average age of 8 years. Similarly, Neyestani and colleagues [35] observed in their clinical trial that, in addition to increasing the serum 25(OH)D concentrations in children, orange juice fortified with VD3 at a dosage of 100 IU/200 mL had greater acceptability among children compared with milk. Pear juice has been also used as a vehicle for vitamin D fortification. Dima et al. [44] showed that the stability of VD3 incorporated in pear juice through microencapsulation with gum arabic and chitosan microparticles was high, and its physicochemical characteristics were maintained for more than seven days.

In addition, some vegetable-based beverages have been successfully fortified with vitamin D, as is the case with an oat-based beverage fortified with 23 IU/100 mL of VD3 [45]. This observation is not in line with Zhou et al. [46], who reported low bioacessibility (around 20%) for oat- and almond-based beverages fortified with VD3. According to these authors, the aggregation and precipitation of the vitamin-laden micelles in the presence of other components in gastrointestinal fluids, such as calcium ions, may be one of the reasons for this low bioaccessibility. Grant and colleagues [47] reported that the incorporation of VD3 in rooibos tea had high acceptability, without changes in the composition and sensory attributes. The authors suggested the consumption of this fortified beverage as part of the daily diet.

From the information depicted in Table 1, it is observed that vitamin D-fortified beverage formulation is based on three criteria: (i) type and (ii) level of vitamin D incorporated in the beverage; and (iii) the technique adopted for vitamin D delivery in the beverage system. Although some studies used VD2 in the fortification of beverages [37,38,41,42,48,49], VD3 has been the typical vitamer used in most formulations. The preference for VD3 may be because it is reported in the scientific literature that this vitamer is more efficient in maintaining serum vitamin D levels [50,51]. Nevertheless, some studies report that VD2 may be as effective as VD3 [42,52]. For instance, Biancuzzo et al. [42] found no significant differences between the types of vitamin D added in orange juice. The dosage of vitamin D added in the beverages was within the recommended tolerable upper intake level (UL) of 100 µg (4000 IU) per day for adolescents and adults, and 50 µg (2000 IU) for children [9]. The minimum vitamin D value found among the studies was 0.57–0.58 µg (≈23 IU)/100 g of liquid [45] and the maximum added dosage was 10,000 IU/200 mL (providing 140% of the recommended 600 IU/day) [47].

## 3. Fortification Strategies of Vitamin D in Beverages

The success of vitamin D fortification in beverages is widely dependent on the homogenization, stability, and bioavailability of vitamin D in the matrix. These three aspects should be addressed in the design of fortified beverages. Loss of vitamin D is mainly due to oxidation and isomerization during processing and storage and has been observed in various systems fortified with vitamin D. In addition, the deposition of vitamin D inside the packaging materials and its degradation in aqueous food matrix are the main source of its instability in beverages. This change affects the appearance and taste of formulated beverages, hence affecting their acceptability to customers [26]. To face these technological limitations, several techniques have been adopted to improve the delivery of vitamin D in fortified beverages, including direct addition, emulsification, and microencapsulation strategies. Table 2 shows different techniques used to develop beverages fortified with vitamin D; the following section addresses the more important findings of these delivery approaches.

### 3.1. Direct Addition of Vitamin D

The direct addition method is the simplest technique for the fortification of beverages. In this approach, vitamin D is dispersed as fine droplets in water or food-grade organic solvents such as ethanol and then mixed with the beverage to ensure its homogeneous distribution in the matrix [26]. The dispersion of vitamin D in water was identified in Hanson and Metzger’s [34] research. The authors investigated the stability of VD3 in milk and chocolate milk submitted, respectively, to high-temperature short-time (HTST) and ultra-high temperature (UHT) sterilization processes. In the case of milk fortification, concentrations of 2.2 g and 5.5 g VD3 were diluted in 97.8 and 94.5 g of distilled water, respectively. In chocolate milk, the concentrations of 1.41 g and 3.52 g VD3 were diluted in 98.59 and 96.48 g of distilled water, respectively. Before sterilization, 10.5 g of these solutions were added and homogenized (13.8/3.4 Mpa) to milk or chocolate milk to obtain fortification levels of 100 and 250 IU/240 mL. Although the literature reports that vitamin D is a sensitive compound to environmental stress such as oxidation, heat, light, and acid pH [64,65], a high stability of VD3 was observed in both fortified milks, even after exposure to high temperatures and storage time, without changes in sensory properties [34]. In accordance with these results, Kaushik et al. [38] observed a good stability of VD2, added to a mixture of cow’s milk and buffalo milk, during pasteurization, boiling and sterilization, storage, and packaging processes. In this research, 1 g of microencapsulated VD2 was diluted and sonicated for 5 min in 10 mL of water (10,000 IU/mL) and then added to milk to reach a fortification level of 600 IU/L. In contrast to the above studies, Zhang and colleagues [45] observed that the UHT process can lead to a loss of 60% of the fortified VD3 in oat-based beverages. However, in this case, VD3 was directly diluted in the beverage without a pre-dilution in water. VD3 was directly added in other beverages, such as orange juice [35,42,43], rooibos tea [47] and an oat-based beverage [45]. According to [42,43] studies, the direct dilution of vitamin D in orange juice did not affect the quality of fortification, showing an increase in serum vitamin D status. Although the direct addition of vitamin D in beverages presents satisfactory results, overall, the reported studies suggest that the application of encapsulation techniques could be more effective in improving the stability and bioavailability of vitamin D in fortified beverages.

### 3.2. Vitamin D Encapsulation Techniques

**Figure 2:**
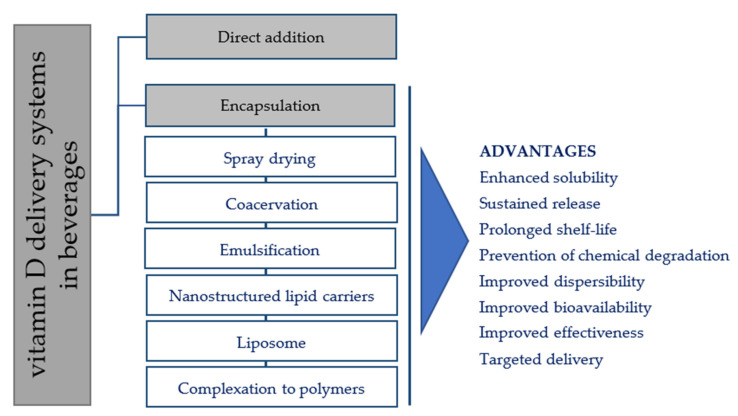
An illustration of various vitamin D delivery approaches used to fortify beverages.

Encapsulation is the technique of producing capsules via insulation of a bioactive core material in a homogeneous or heterogeneous coating matrix [26]. If the particle sizes (PS) of capsules are about 1–5000 μm, they are called microcapsules [10]; colloidal-sized particles with diameters ranging from 10 to 1000 nm are referred as nanocapsules [66]. Vitamin D microencapsulation has been widely used in the preparation of fortified beverages and offers several advantages in comparison to the direct addition and emulsification approaches (Figure 2). Some advantages include: (i) higher solubility barrier between vitamin D and the food matrix; (ii) better homogeneity in the dispersion (iii) higher bioavailability of vitamin D though the controlled and targeted release of the encapsulated vitamin; and (iv) improved physiochemical (e.g., moisture, oxidation, pH, temperature, mechanical) and organoleptic characteristics (e.g., appearance, taste, quality of food matrix) of the final products [8,53]. However, although many of the microencapsulation systems developed for vitamin D delivery look promising, so far, applications have not been found at the industrial level. Spray-drying and coacervation are the two usual methods for microencapsulation of vitamin D in beverage products [67]. Other microencapsulation techniques with great potential in the design of vitamin D-fortified beverages include micro/nanoemulsions, spray drying, nanostructured lipid carriers, liposomes, and polymer complexation; these approaches will be discussed in the following subsections. Several parameters, namely pH, ionic strength, and heat treatments, can potentially affect these delivery systems [68], being essential to test their stability during food processing.

#### 3.2.1. Spray Drying Technique

Spray drying is the oldest technique used for bioactive compound encapsulation due its low cost, easy scale-up and great flexibility in the choice of wall materials [69]. In this technique, vitamin D is homogenized in a dispersion containing polymeric wall material, then the homogenized dispersion is fed to the spray dryer and atomized by hot air that leads to the development of nanomaterials as consequence of water evaporation [26,69]. Although spray drying is a common technique, process limitations result in porous nanomaterials that are prone to cause the degradation of the encapsulated vitamin D. This limitation explains the low applicability of vitamin D encapsulation in beverage fortification. To date, three reports are documented using the spray drying technique to encapsulate vitamin D in beverage systems [36,44,70]. Lovett [70] produced microcapsules of VD3 with bovine β-lactoglobulin (β-Lg) via a spray drying process and used them for the enrichment of sports drinks. The bioavailability of VD3-fortified drinks was assessed in female Wistar rats at three levels (10, 20, and 40 µg/L). After a 6-week supplementation treatment of 40 μg VD3/day, rats receiving the VD3-β-Lg complex had serum 25(OH)D levels significantly greater (*p* < 0.0001) than the nonencapsulated VD3 group. Khaganate et al. [36] used spray drying to deliver VD3 in milk. Results showed that the delivery system of VD3 was an effective and safe strategy in improving serum 25(OH)D levels in children aged 10–14 years. More recently, Dima et al. [44] prepared pear juice fortified with VD3 loaded in gum arabic–chitosan microparticles using a spray drying technique. In this study, VD3 loaded in gum arabic–chitosan microparticles had an average diameter of 12.64 ± 1.14 μm and an encapsulation efficiency (EE) of 89.78 ± 3.88%; the pear juice fortified with VD3-loaded in gum arabic-chitosan microparticles remained unchanged after seven days of storage at 4 °C. The selection of adequate wall materials and the combination of the spray drying technique with other microencapsulation approaches could effectively address the stability issues of vitamin D in beverage systems.

#### 3.2.2. Coacervation Technique

In the coacervation technique, also known as phase separation, two or more electrolytes with opposite charges are mixed under suitable conditions forming two liquid phases, a diluted polymer phase that acts as a continuous medium and a polymeric solution involved in the encapsulation system [10]. Proteins and polysaccharides are the most used biopolymers in the coacervation process [67]. The ability of the coacervation technique to encapsulate and protect vitamin D in beverage systems is still unexploited, being used in only two research works till date. In the first report, Jannasari et al. [10] encapsulated VD3 using cress seed mucilage and gelatine, and its physicochemical properties and in vitro and in vivo behaviours were investigated. The optimum microcapsules presented a PS of 137.22 ± 3.21 μm and an EE and loading capacity (LC) of 67.93 and 50.9%, respectively. The in vivo test performed on male rats confirmed the efficiency of microencapsulated VD3 in increasing the 25(OH)D serum levels. In the second report, Santos et al. [53] prepared a gelatine A and carboxymethyl tara gum coacervate for the encapsulation of VD3. The greatest EE (80%) and PS (0.25 μm) were achieved at a total concentration of biopolymers of 1%, a ratio of 1:2 and pH of 4.0; the in vitro gastrointestinal simulation showed a more pronounced release in the small intestine with a VD3 bioaccessibility of 56%.

#### 3.2.3. Emulsification Technique

Emulsions are colloidal dispersions of two immiscible phases used to encapsulate, protect, and deliver lipophilic components [71]. They are categorized as coarse emulsions, microemulsions and nanoemulsions based on their droplet size and stability [72]. Microemulsions differ from nanoemulsions because they are thermodynamically stable colloidal systems [73]. There are two types of emulsions, the oil-in-water (o/w) or water-in-oil (w/o), depending on whether the dispersed phase is the oil or water phase [13,74]. The production of emulsions requires a water phase, an oil phase, emulsifiers, surfactants, and input of either high- (e.g., microfluidics, high pressure homogenization or ultrasounds) or low-energy methods (e.g., spontaneous emulsification, emulsion phase inversion, phase inversion temperature) [72]. High-energy methods are preferred at the industrial scale to produce large amounts of emulsions [29].

Emulsions are good delivery systems for encapsulating vitamin D into beverages due to their transparent appearance. However, the development of stable emulsions in this type of matrix has some challenges, mostly related to the difficulty of homogenizing vitamin D, the limited availability of food-grade emulsifiers and the chemical degradation of vitamin D under heat treatment and storage conditions [29]. Some examples of applications for the encapsulation of vitamin D in beverage products are summarized in Table 2; information about the main ingredients (emulsifiers, oil, and aqueous phase composition) and the physico-chemical attributes of the droplets is included. Various types of food grade surfactants and emulsifiers have been used in the preparation of emulsions for vitamin D delivery in beverages. Some examples of emulsifiers include higher molecular weight biopolymers, such as amphiphilic proteins (casein, lactoferrin, β-Lg, protein isolates), and polysaccharides (gum arabic, modified starch, cellulose). Different mechanisms, such as Ostwald ripening, gravitational separation, coalescence, and droplet flocculation tend to destabilize emulsions during storage. To address this technological limitation, single or mixed surfactants are added to attain the long-term stability of emulsions in beverages [48]. Examples of surfactants used in the preparation of vitamin D-fortified beverages include small molecules (Tween 20, 80, Poloxamer 407), phospholipids (lecithin, canola/corn/soybean oils, precirol, compritol, miglyol), glycosides (quillaja saponin, pectins, chitosan), and proteins (pea protein, gelatine).

Golfomitsou et al. [54] prepared a Tween 20–soybean lecithin nanoemulsion as a carrier of VD3 to fortify full-fat milk. The nanoemulsions were prepared by High Pressure Homogenization (HPH), using Tween 20 and soybean lecithin as emulsifiers, and soybean oil or a mixture of soybean oil and cocoa as the oil phase. The VD3-enriched nanoemulsion with a PS < 200 nm was incorporated into full-fat milk to obtain a product enriched with 0.05 µg VD3/mL. Data showed that the PS of the milk emulsion was not influenced by the presence of the loaded nanoemulsion, being stable for a minimum of 10 days of storage. Mehmood et al. [48] prepared VD2 nanoemulsions using a mixed surfactant-based approach (Tween 80 and soya lecithin) and ultrasonic homogenization techniques. The nanoemulsions with a PS < 200 nm remained stable against a wide range of temperatures (30–90 °C), pH values (2–8), ionic strengths (50–400 mM), and four freeze-thaw cycles. After 30 days of storage, VD2 retentions were 74.4 ± 1.2 and 55.3 ± 2.1% in nanoemulsions stored at 4 and 25 °C, respectively. Zhou et al. [46] examined the impact of organic (nanocellulose) and inorganic (TiO_2_) nanoparticles on the physicochemical properties and bioavailability of VD3-fortified almond and oat milks. Quillaja saponin was used as a natural plant-based surfactant and the plant-based milks were fortified with 10% vitamin D-loaded nanoemulsions. The authors observed that nanocellulose was most effective at increasing the shear viscosity of the fortified plant-based milks, whereas the TiO_2_ nanoparticles were more effective at increasing the whiteness property. Both fortified milks exhibited low bioaccessibility of VD3, which was around 20%. Also, a pea protein nanoemulsion was evaluated as a novel vehicle for the fortification of several beverages, namely lower-fat cow milk, canned orange juice, orange juice powder, banana milk, and infant formula [75]. Nanoemulsions were prepared by pH-shifting and ultrasonication combined techniques and formulations were tested by sensory evaluation regarding taste and palatability. The formulated nanoemulsions with a PS of 21.8 nm successfully enhanced the VD3 content in all fortified beverages, with protection from UV degradation and without affecting the colour, viscosity, chemical composition, or antioxidant activity. The emulsion structures can be affected by chemical and physical instability mechanisms [13]. Although it is well-reported in the literature that the presence of an antioxidant in the emulsion system enhances the stability of Vitamin D in the fortified beverages [26], this parameter has been scarcely evaluated.

#### 3.2.4. Nanostructured Lipid Carriers (NLC)

Nanostructured lipid carriers (NLC) are unstructured solid-lipid matrices comprised of a mixture of liquid and solid lipid blend and an aqueous phase consisting of a surfactant or a mixture of surfactants [28,76]. Typically, the liquid and solid lipids are blended in a ratio that could vary from 70:30 to 99.90:0.10, while the surfactant content is kept between 1.5–5% (*w*/*v*) [26]. Despite its promising applicability, it is still an unexplored method for vitamin D encapsulation in the design of fortified beverages. Phase inversion-based cold water dilution and hot homogenization have been the preferred techniques to prepare NLC for vitamin D delivery in fortified beverages (Table 2). For instance, Mohammadi et al. [28] applied the hot homogenization technique to prepare various formulations with different concentration of precirol (2.92–4% *w*/*v*), compritol (2.92–4% *w*/*v*), miglyol (0.4–1.48% *w*/*v*) and the surfactants tween 20 (2–6% *w*/*v*), Tween 80 (1–6% *w*/*v*) and poloxamer 407 (1–6% *w*/*v*), either alone or in combination. The authors observed that the PS and size distribution (span value) of the formulations were greatly dependent on the formulation compositions. Overall, the precirol-based NLC showed higher physical stability compared to compritol-based NLC, and the intestinal absorption of VD3, tested in male Wistar rats, was enhanced by incorporating NLCs. Seo et al. [55] also applied the homogenization technique to prepare NLCs for vitamin D delivery in fortified beverages. These authors evaluated the effects of different carrier oils and the use of the emulsifier polyglycerol polyricinoleate (PGPR) on the stability of the VD3 emulsions. Compared to glyceryl monostearate (GMS), used as a control, PGPR resulted in relatively small PS (0.30 to 0.43 μm) and high EE (85.2% to 90.4%). The stability of the NLC emulsions against environmental stresses was also evaluated under varying conditions of ionic strength, pH, freeze-thaw cycles, and storage at different temperatures. When compared to GMS, PGPR emulsions were stable at high ionic strengths but unstable at pH < 3 and showed higher liquid dispersion stability at 25 and 65 °C [55]. Like the work of Mohammadi et al. [28], the formulated NLCs were not tested in real beverage systems. Maurya et al. [39] developed a phase inversion-based cold water dilution method to VD3 delivery in a milk-based beverage. The optimal encapsulation formulation for the fortification of a milk-based beverage ‘Lassi’ was 20% (*v*/*v*) Kolliphor, 20% (*v*/*v*) caprylic-/capric triglyceride (CCGT), 60% (*v*/*v*) water, 2.5% (*w*/*v*) leciva, 2% (*w*/*v*) VD3 and 5% (*w*/*v*) sodium chloride. The high VD3 stability under environmental stress conditions (temperature and humidity, pH, and ionic strength) and sensorial acceptability confirmed the suitability of the developed NLC for the purpose of fortification of VD3 in this type of beverage.

#### 3.2.5. Liposome

Like nanoemulsions and NLCs, liposomes are lipid-based carriers with the ability to encapsulate vitamin D in beverage systems [56]. These systems, also called nanoscale lipid bilayers or submicron bilayer lipid vesicles, are spherical or oval vesicles consisting of a phospholipid bilayer entrapping a central liquid core [77,78]. They are formed by the dispersion of phospholipid molecules in a water-based medium by energy input, which could be physical (e.g., thin-film hydration, ethanol/ether injection, reverse phase evaporation, microfluidisation), mechanical, thermal, acoustic (e.g., ultrasonication), or a combined technique [74,78,79]. Although liposomes allow high chemical stability of vitamin D, their application in beverage fortification is still not fully explored. To our best knowledge, only one work used liposomes to encapsulate vitamin D in beverages [56]. Mohammadi et al. [56] prepared a VD3 nanoliposome applying a thin-film hydration–sonication technique. The formulated liposome had an EE higher than 93%, and PS and span value mean ranges of 82–90 nm and 0.70–0.85, respectively. The addition of cholesterol to lecithin bilayer increased the negative zeta potential (ζ-potential) from −29 to −43 mV.

### 3.3. Vitamin D Polymers Complexation

Several protein-based nanocarriers have been developed as vehicles of vitamin D in fortified beverages. Milk proteins such as casein [37,57,58,59], whey [60], α-lactoalbumin (αLa) [63] and β-lactoglobulin (βLg) [49,61], have been the most frequently involved proteins to deliver vitamin D in beverages systems. Reassembled casein micelles (rCMs) have been reported to enable the vitamin D fortification of milk products with minimum impact on the functional behaviour of caseins during processing and on sensory attributes of milk products [57]. Haham et al. [57] prepared vitamin-D-loaded rCMs by Ultra-High-Pressure Homogenization (UHPH) (~155 MPa) with a PS of 91 ± 8 nm. The developed rCM better protected VD3 encapsulated against thermal degradation and during cold storage compared to the unencapsulated or to Tween-80-emulsified VD3. The bioavailability of the system delivery was investigated in a randomized double-blinded placebo controlled clinical study with human volunteers. Results showed that a single dose of 50,000 IU VD3 encapsulated in rCMs (in 1% fat milk) increased 25(OH)D serum levels in comparison to the control, an aqueous Tween-80-emulsified VD3 supplement. A recent work was also carried out on the preparation of various VD2–sodium caseinate complexes and the evaluation of their stability under different storage temperatures [37]. The fortified milk with VD2–sodium caseinate complexes was subjected to different heat treatments commonly used in the dairy industry (pasteurization, boiling and sterilization) and evaluated for the stability of VD2. Results showed that VD2–sodium caseinate complexes (NaCas–VD, SNaCas–VD, RNaCas–VD and RSNaCas–VD) and free VD2 showed the highest stability when stored at −20 °C, followed by 4 °C and 37 °C. Moreover, caseinate–VD2 complexes provided greater stability to withstand the heat treatments.

Besides caseins, other bovine milk proteins have been used to entrap VD3 for fortification. For instance, Abbassi et al. [60] encapsulated VD3 in whey protein nanoparticles and evaluated its stability in the presence of air for 7 days. The complexation of VD3 with whey proteins proved to increase the stability of the vitamin under light and prolonged storage, compared to the free form. The authors suggested that the VD3–whey protein complex could be used for the enriching of clear or non-clear drinks such as herbal beverages and fruit drinks, but no data obtained on real foods were given. Furthermore, Diarrassouba et al. [61] prepared a βLg–VD3 complex and evaluated its stability upon exposure to normal and extreme conditions over time, simulated gastrointestinal digestion and permeability through the intestinal cells, as well as in vivo efficacy using an animal experiment. Data showed that the VD3–βLg complex (i) significantly improved the stability of VD3 at 4 °C and when exposed to UV light; (ii) was resistant to proteases in simulated GI digestion, and (iii) crossed the Caco-2 cells monolayers, which was confirmed by the significant increase in the concentration of 25(OH)D in rats fed with the βLg–VD3 complex compared to the ones fed with the free VD3. The authors suggested that the fortification of milk products with the βLg–VD3 complex can be recommended to improve the intake of this vitamin.

As alternatives to milk proteins, plant-based proteins have been proposed as potential nanovehicles for the delivery of vitamin D in beverages. For instance, the nanocomplexation of VD3 with potato proteins provided significant protection and reduced vitamin losses during pasteurization and simulated shelf-life tests under several different sets of storage conditions [6]. Moreover, results showed that the VD3–potato protein complex may be suitable for the enrichment of clear beverages. Likewise, polysaccharides complexed to proteins have been proposed as promising approaches to deliver vitamin D in beverages systems. Ron et al. [49] prepared a VD2–βLg–low methoxyl pectin complex as a suitable nanovehicle for VD2 incorporation into clear beverages. The authors reported that the nanocomplex provided higher protection against VD2 degradation and stability compared to the unprotected vitamin dispersion. The system was transparent and suitable for the enrichment of clear acid beverages. Another interesting approach was recently presented by Lamsem et al. [7]. These authors investigated gum arabic as a VD3 carrier for incorporation of VD3 into beverages; the results suggested that VD3 was successfully encapsulated using gum arabic with the highest LC of 3.47% and the lowest EE of 61.24%. The VD3–gum arabic complex exhibited good stability across a pH range between 2.0 and 7.4 during 100-day refrigerated storage, and a bioaccessibility significantly higher (95.76%, *p* < 0.05) than the nonencapsulated VD3 (68.98%). During 2-week supplementation of 60 μg VD3/day, rats receiving the VD3-gum arabic complex had 25(OH)D levels of at least 81 ng/mL higher than that of the nonencapsulated VD3 group.

Another approach to the vitamin D fortification of beverages is the development of liprotides, i.e., complexes between partially denatured proteins and lipids. In this system, the protein forms a stabilizing shell around a fatty acid micelle core [63]. Pederson et al. [63] evaluated different liprotides consisting of different cis fatty acids with variable chain lengths and saturation (oleate, linoleic acid, cis-palmitoleic acid, cis-vaccenic acid and eicosenoic acid) and whey proteins (bovine serum albumin, αLa and βLg), and evaluated their ability to protect VD3 upon exposure to heating or intense UV light. Results showed that the formed liprotides are water-soluble, transparent, protect VD3 against elevated temperatures and UV light, and were stable at neutral pH. Besides its suitable use as a means of vitamin D delivery in beverages, its applicability to real foods was not evaluated.

Encapsulating vitamin D by one or more encapsulation techniques is the best approach to achieve the desired stability, bioavailability and dispersibility of vitamin D in fortified beverages. Even though many of the developed carrier systems for vitamin D delivery in fortified beverages (as reported in Table 2) seem to be promising, they have not found application at the industrial level so far. Thus, advanced knowledge on the process optimization under industrial conditions should be promoted.

## 4. Stability, Bioaccessibility, and Bioavailability of Vitamin D-Fortified Beverages

The stability of vitamin D, i.e., its ability to tolerate the effects of pH, ionic strength, and temperature changes, is a critical parameter to consider in the formulation of a vitamin D-fortified beverage. The cis-triene configuration of vitamin D (Figure 1) makes it sensitive to isomerization and oxidation. As this phenomenon does not involve the side chain of VD2 and VD3 [13], both vitamers have been used for the fortification of beverages. High stability of vitamin D in fortified milks has been documented in the literature. For instance, Khadgawat et al. [36] reported a 10% VD3 loss in fortified milk after 12 weeks of storage. Although the literature reports that vitamin D is a sensitive compound to environmental stress such as oxidation, heat, light, and acid pH [13,65], Hanson and Metzger [34] observed a good VD3 stability in fortified (by direct addition) milk and chocolate milks, even after exposure to high temperatures (73 and 138 °C) and storage time (60 days), without changes in sensory properties. Kaushik et al. [38] observed a good stability of VD2 added to a mixture of cow and buffalo milk during pasteurization, boiling and sterilization, storage, and packaging processes. Likewise, Syama et al. [37] evaluated the stability of VD2 (free and in complexation with casein) in a UHT 3% and 8.5% fat milk under different storage conditions. Authors found that the temperature and type of material can influence the stability of VD2-fortified milk, showing that glass bottles are a more suitable packaging material compared to low-density polyethylene pouches, and that the greatest stability was at −20 °C and the lowest stability at 37 °C. The stability of VD3 was also studied in “Lassi” milk-based beverage. Maurya and Aggarwal [39] evaluated the stability of VD3 added through the NCL method and observed high physicochemical stability against temperature, pH, and ionic strength. Fauziyyah et al. [40] observed that fermentation time in goat’s-milk kefir can influence the stability of VD3; the highest level of VD3 was found after 6 h of fermentation. Like milk, fortified juices present good stability of vitamin D. Tangpricha et al. [41] observed that VD3 fortified in orange juice remained stable for a period of 30 days of storage at 4 °C. Dima et al. [44] used the VD3–gum arabic–chitosan complex to fortify the pear juice and evaluated the stability of VD3 for a period of 7 days of storage at 4 °C. Authors suggested that, in addition to the microencapsulation method, the good stability of VD3 in the fortified pear juice could be attributed to the presence of natural antioxidant compounds, namely flavanoids and vitamin C. Plant-based beverages fortified with vitamin D have showed low stability of this vitamin. Zhang et al. [45] fortified oat-based beverages with VD3 and reported a 60% loss of vitamin D content during processing. The authors justified that VD3 can react with a certain compound that existed in limited amounts in the liquid matrix, and that after the depletion of this compound, the remaining VD3 was not affected.

The bioavailability of vitamin D is influenced by three important phenomena: (i) bioaccessibility, i.e., vitamin release from the food matrix after in vitro digestion; (ii) transformation, i.e., chemical, and biochemical conversion; and (iii) absorption, i.e., migration through the mucus layer to the surfaces of the intestinal lumen and uptake by epithelial cells [13]. Zhou et al. [46] prepared almond and oat milks fortified with VD3 in O/W nanoemulsion made with corn oil, and VD3 nanoemulsion with added TiO_2_ (inorganic) or nanocellulose (organic). For all the fortified plant-based milks, the VD3 bioaccessibility was around 20%, indicating that the type of nanoparticle (inorganic or organic) had no impact on the bioaccessibility of VD3. The reasons for the observed low bioaccessibility were hypothesized to be either a saturation of the mixed micelles or the aggregation and precipitation of the majority of VD3-loaded mixed micelles due to some of the constituents in the plant-based milks. Lamsen et al. [7] evaluated the encapsulation of VD3 in gum arabic for beverage applications and observed a high bioaccessibility (95.76%) compared to nonencapsulated VD3 (68.98%), and an increase in 25(OH)D levels (>81 ng/mL) after 2-week supplementation (Sprague-Dawley rats) with 60 μg VD3/day. However, it is worth noting that the bioaccessibility of VD3 was not evaluated in the food matrix.

Studies of the bioaccessibility of vitamin D fortification in beverages are still scarce and not well elucidated. However, intervention studies have been conducted indicating that vitamin D-fortified milk and orange juices have good bioavailability in humans. Haham et al. [57] incorporated VD3 in casein micelles and observed a high bioavailability of 1% fat milk fortified with 50,000 IU of VD3 in 87 human volunteers. Khadgawat et al. [36] reported that 200 mL of milk fortified with 600 IU (15 μg) or 1000 IU (25 μg) of VD3 is effective in improving the 25(OH)D serum levels in children aged 10 to 14 years. Neyestani et al. [35] showed that fortified milk (200 mL) with 100 IU VD3 increased the 25(OH)D serum levels of children aged 10 to 12 years. Economos et al. [43] observed that a multinutrient-fortified juice improved the vitamin D status in children; Tangpricha et al. [41] reported that ingestion of orange juice fortified with 1000 IU of VD3 for 12 weeks can increase serum 25(OH)D concentrations by up to 150% in adults, and Biancuzzo et al. [42] found that fortification of orange juice with VD2 or VD3 is as effective as an oral supplement in maintaining vitamin D status.

## 5. Regulation of Vitamin D-Fortified Beverages

Vitamin D (VD2 or VD3, in crystalline, resin or crystal form) is Generally Recognized as Safe (GRAS) at specified maximum levels of safe use for certain beverage products and infant formula [7]. Some vitamin-D fortified beverages are currently sold in several countries, namely USA, Canada, Finland, and Australia (Table 3).

In USA, the fortification of beverages with vitamin D is regulated by the Food and Drug Administration (FDA) [81]; manufacturers are allowed to voluntarily add up to 84 IU/100 g of VD3 to milk, 100 IU/240 mL of VD3 to calcium-fortified fruit juices and fruit juice drinks, 50 IU/100 g of soy beverages, 84 IU/100 g of VD2 to plant-based beverages and 89 IU/100 g of VD2 to plant-based yogurt alternatives. In Europe, the legal policy responsible for vitamin D fortification of beverages is Annex 1 of Regulation (EC) No 1925/2006, amended by the Commission Regulation (EC) No 1170/2009 [82].

## 6. Concluding Remarks

The present review gives an insight regarding current advances in the technological aspects of fortifying beverages with vitamin D. To date, several beverages have been fortified with vitamin D, such as milk, fruit juices, tea, and vegetable drinks. Encapsulation seems to be an indispensable tool to design vitamin D materials with the desired functionality to deliver vitamin D through beverages, with advantages over the direct addition and emulsification approaches. Some advantages of the encapsulation delivery systems include improved stability against light exposure, chemical and mechanical stress, better homogeneity with the matrix, improved oral bioavailability, and improved organoleptic properties. Although these advantages exist, there are still improvements needed to increase the efficacy of these vitamin D delivery systems in beverages. For example, they should be formulated with natural and safe ingredients, they should not adversely affect the sensory and quality attributes of the developed beverages, they should prevent vitamin D from chemical degradation with a longer shelf-life, and they should provide increased bioavailability. There is limited information on the in vivo bioavailability studies of beverages fortified with vitamin D. Hence, in vivo studies supported with interdisciplinary knowledge should be conducted to ascertain the effectiveness of these delivery systems to develop vitamin D-fortified beverages and address vitamin D deficiency disease.

## Figures and Tables

**Table 1:** Fortification approaches of vitamin D in different types of beverages.

Fortified Beverage	Country	Formulation	Fortification Level	Processing	Vitamin Stability and Bioaccessibility	Effects on Sensory Properties	Effects on Health	Ref.
HTST 2% fat milk	USA	Water dispersible VD3	250 IU/240 mL	HTST (73 °C for 15 s) and storage at 4 °C for 21, 42 and 60 days	Tolerate HTST No loss of VD3 during storage at 4 °C	No significant changes in composition and sensory attributes	NE	[34]
UHT 2% fat chocolate milk	USA	Water dispersible VD3	100 IU/240 mL	UHT (138 °C for 2 s) and storage at 4 °C for 21, 42 and 60 days	Tolerate UHT No loss of VD3 during storage at 4 °C	No significant changes in composition and sensory attributes	NE	[34]
Milk	Iran	ND	100 IU/200 mL	NE	NE	Lower acceptance compared to orange juice	↑[25(OH)D] serum levels	[35]
Milk	India	VD3 Spray Drying	600 IU or 1000 IU/200 mL	NE	Stability loss <10% after 12 weeks of storage period	NE	↑[25(OH)D] serum levels	[36]
UHT 3% and 8.5% fat milk	India	VD2-protein complexes (NaCas-VD, SNaCas-VD, RNaCas-VD and RSNaCas-VD)	500 IU/L	Pasteurization (63 °C/30 min), boiling and sterilization (121 °C for 15 min at 15 psi)	Higher stability during storage at −20 °C, followed by 4 °C and 37 °C	NE	NE	[37]
Cow and buffalo milk	India	VD2 Encapsulation	600 IU/L	Pasteurization (63 °C/30 min), boiling and sterilization (121 °C for 15 min at 15 psi)	Stable during pasteurization, boiling, sterilization, packaging, and storage conditions	NE	NE	[38]
“Lassi” milk-based beverage	India	VD3-NLC	400 IU/100 mL	Environmental stress conditions of temperature and humidity, pH, and ionic strength	High physicochemical stability against temperature, pH, and ionic strength	No significant changes in composition and sensory attributes	NE	[39]
Goat milk kefir	Indonesia	VD3	42 IU/100 mL	Pasteurization at 72 °C for 15 s and cooling to 25 °C Different times of fermentation tested: 0, 6, 12, 18, and 24 h	The highest level of VD3 was found after 6 h of fermentation	Higher viscosity after 24 h of fermentation	NE	[40]
Orange juice and milk	USA	VD3	1000 IU/240 mL	NE	No loss of VD3 during 30 days of storage at 4 °C. The fat content of milk did not affect the bioavailability of VD3	NE	↑[25(OH)D] serum levels	[41]
Orange juice	USA	Water dispersible VD3 or VD2	1000 IU VD3 or VD2/240 mL orange juice or capsule	NE	VD2 and VD3 were equally bioavailable in orange juice and capsules	NE	↑[25(OH)D] serum levels	[42]
Orange juice	USA	ND	100 IU/240 mL	NE	NE	NE	↑[25(OH)D] serum levels	[43]
Orange juice	Iran	ND	100 IU/200 mL	NE	NE	Higher acceptance compared to orange juice	↑[25(OH)D] serum levels	[35]
Pear juice	Romania	VD3-gum arabic- chitosan complex Spray drying	0.002 g/100 mL	NE	No loss of VD3 during 7 days of storage at 4 °C	NE	NE	[44]
Oat-based beverage	Sweden	VD3	23 IU/100 g of liquid	Sterilization at 140 °C for 5 or 20 s	Stability loss of 60%	NE	NE	[45]
Almond and oat milks	ND	VD3 nanocellulose or TiO_2_ nanoemulsion	0.4 wt%	NE	Low bioaccessibility (~20%) of VD3 loaded in VD3-nanocellulose or TiO_2_ nanoemulsion	Nanocellulose increased the shear viscosity, while TiO_2_ particles increased the whiteness of fortified milks	NE	[46]
Rooibos Tea	Canada	Water dispersible VD3	10,000 IU/200 mL	NE	ND	No significant changes in composition and sensory attributes High sensorial acceptance	NE	[47]

Legend: not defined (ND); not evaluated (NE); seconds (s); ↑ (increase); nanostructured lipid carrier (NLC); ultra-high temperature (UHT); high-temperature short-time (HTST); sodium caseinate complex (NaCas-VD); succinylated sodium caseinate complex (SNaCas-VD); Reassembled sodium caseinate–vitamin D2 complex (RNaCas-VD); Reassembled succinylated sodium caseinate–vitamin D2 complex (RSNaCas-VD).

**Table 2:** Techniques adopted for development of vitamin D-fortified beverages.

Technique	Preparation Method	Matrix Composition	Physico- Chemical Attributes	Fortification Level in Beverage	Main Observations	Ref.
Coacervation	Microencapsulation (VD3-cress seed mucilage–gelatine complex)	Optimum conditions: -core to shell ratio: 0.76 cress seed mucilage-to -gelatine volume ratio: 0.36; pH 3.4	PS (μm) 137.22 ± 3.21 EE (%) 67.93 LC (%) 50.9	NE	28 and 70% VD3 delivery to gastric and intestinal media after 2 and 6 h, respectively Increase of body height, weight and 25(OH)D serum levels in male albino rats (6-week treatment)	[10]
	Microencapsulation (VD3-carboxymethyl tara gum– gelatine A complex)	Optimum conditions: core to shell ratio: 1:2; carboxymethyl tara gum- gelatine A ratio: 6; pH 4.0	PS (μm) 0.25 EE (%) 80	NE	Bioaccessibility of 56% after in vitro digestion	[53]
Nanoemulsion	High pressure homogenization (VD3-tween 20-soybean lecithin complex)	0.8% (*w*/*w*) VD3 90% (*w*/*w*) water 4% (*w*/*w*) tween 20/ lecithin (3:1) 6% (*w*/*w*) soybean oil	Two populations of droplets: PS (nm) 146 ± 7 due the presence of surfactant micelles PS (nm) 21 ± 1 due the presence of micelles	Whole-fat milk 600 IU VD3/250 mL	Droplet diameter and PS of milk were not affected by the presence of the O/W nanoemulsion The fortified milk was stable under particle growth and gravitational separation for at least 10 days	[54]
	Ultrasonic homogenization (VD3-tween 80-soybean lecithin complex)	5% VD2 8% (*w*/*w*) canola oil 3% (*w*/*w*) tween 80 1% (*w*/*w*) soybean lecithin	PS (nm): <200	NE	PS of 140.15 nm (4 °C) and 155.5 nm (25 °C) Stability of 74.4% and 55.3% (30 days storage at 4 °C and 25 °C, respectively)	[48]
	Blend of the oil phase (10% *w*/*v*) and the aqueous phase (90% *w*/*v*), followed by microfluidization	60% (*w*/*w*) corn oil 40% (*w*/*w*) VD3 1% (*w*/*w*) quillaja saponin 1% (*w*/*w*) nanocellulose/TiO_2_	PS (nm): 140 (nanocellulose); 600 (TiO_2_) ζ-Potential (mV): -39.4 ± 3.2 (nanocellulose); −35.0 ± 1.3 (TiO_2_)	Almond and oat milks 10% VD3 (*w/v*)	TiO_2_ nanoparticles were most effective at increasing the whiteness of the fortified milk, whereas the nanocellulose ones were most effective at increasing the shear viscosity Low VD3 bioaccessibility (≈20%)	[46]
Nano- structured lipid carrier (NLC)	Hot homogenization technique	VD3 2.92–4% (*w*/*v*) precirol 2.92–4% (*w*/*v*) compritol 0.4–1.48% (*w*/*v*) miglyol 2–6% (*w*/*v*) tween20 1–6% (*w*/*v*) tween80 1–6% (*w*/*v*) poloxamer407	PS (nm): 77–2504 Span value: 0.77–3.65	NE	An optimum concentration of 3% of Poloxamer407 or 2% of Tween20 was sufficient to prevent agglomeration during the homogenization process VD3 intestinal absorption was enhanced by incorporating NLCs	[28]
	Phase inversion-based cold water titration method	20% (*v*/*v*) kolliphor 20% (*v*/*v*) CCTG 60% (*v*/*v*) water 2.5% (*w*/*v*) leciva 2% (*w*/*v*) VD3 5% (*w*/*v*) sodium chloride	PS (nm): 48.61 ± 1.58 ζ-Potential (mV): −17.310 ± 0.501 EE (%): 96.82 ± 0.31 VD3 release (%): 22.54 ± 0.33	Lassi 400 IU VD3/100 mL	High stability under different environmental stress conditions (temperature, pH, and ionic strength) Higher level of sensorial acceptability compared to control	[39]
	Hot homogenization technique	100 mg VD3 3 g soybean lecithin 2.5 g MCT oil 4 g GMS or PGPR 5 g of Poloxamer	PS (nm): 300–430 ζ-Potential (mV): −39.5 to−67.8 EE (%): 85.2−90.4	NE	Higher VD3 stability under different environmental stress conditions (temperature, pH, and ionic strength) compared to control	[55]
Nanoliposomes	Thin film hydration–sonication technique	60:0, 50:10, 40:20, 30:30 (*w*/*w*) mixtures of lecithin and cholesterol 15 mL (2:1 *v*/*v*) EOH/MeOH 10 mL distilled water	PS (nm): 82–90 Span value: 0.70–0.85 ζ-Potential (mV): −29 to −43 EE (%): 93	NE	High protection against VD3 degradation	[56]
Polymer complexation	Ultra-high-pressure homogenization (VD3–casein complex)	162.5 mg/mL VD3 solution 1.25% (*v*/*v*) EtOH 10 mg/mL caseins 0.009 M K_2_HPO_4_ 0.004 M tri- potassium citrate 0.011 M CaCl_2_	PS (nm): 91 ± 8	1% fat milk 50 000 IU VD3/100 mL	High stability during thermal treatment (80 °C, 1 min) and 28-day cold storage (≈10%) compared to Tween-80-VD3 complex and unencapsulated VD3 -Increased 25(OH)D serum levels in humans	[57]
	Ultra-high-pressure homogenization (VD3–casein complex)	6.11 mg/mL sodium caseinate 8 mg/mL VD3 solution 1.2% EOH	PS (nm): 95 ± 2 –89 ± 0.3	NE	High stability in gastric and upper-intestinal conditions, High bioavailability in vitro	[58]
	Vortex stirring for 30 s at room temperature (VD3–casein-maltodextrin complex)	0.02% (*w/w*) casein in water 0.25% (*v*/*v*) EOH	PS (nm): <30 nm EE%: 90%	NE	Provide protection against degradation at low pH, and during shelf life at neutral pH and 4 °C	[59]
	Add dropwise, homogenization and freeze-drying (VD2–sodium caseinate complex)	VD2-casein-complexes: -VD2-NaCas -VD2-SnaCas -VD2-RnaCas -VD2-SNaCas		Milk 500 IU VD2/1000 mL	Stability up to 78.9% (−20 °C), 74.0% (4 °C) and 21.4% (37 °C) Higher stability for VD2-casein complexes and free-VD2 fortified milk stored in transparent glass bottles upon exposure to different light intensities VD2 stability of 90 and 67% when submitted to pasteurization (63 °C/30 min), boiling and sterilization (121 °C/15 min/15 psi) treatments, respectively	[37]
	Girox method (VD3–whey protein isolate complex)	8% (*w*/*w*) WPI solution 54 mg VD3/100 mL WPI solution 50 mM CaCl_2_	PS (nm): 80.0–260	NE	VD3 should be added to WIP solution before pH cycling. Presence of CaCl_2_ in nanoparticle composition reduces VD3 degradation during storage time. WPI–VD3 nanoparticles can be used for enriching of clear or non-clear drinks	[60]
	Homogenization (VD3–βLg complex)	0.2% β-lactoglobulin solution 10 mg VD3 in 25 mL MeOH (2:1 βLg/VD3 complex)	VD3 release: 24.5 ± 0.73% and 40.9 ± 0.71% (absence and presence of pancreatin, respectively)	NE	Increased VD3 stability at 4 °C and UV light exposure Resistance to proteases in simulated GI digestion Increased 25(OH)D levels in rats fed with β-lactoglobulin-VD3 complex	[61]
	Add dropwise and vortexing (VD3–potato protein isolate complex)	Different concentrations of VD3 1 mg/mL potato protein stock solution (79 μM)	PS (nm): −33 to −116	NE	Stability under different environmental stress conditions (during pasteurization, shelf life) Maintain optical clarity in aqueous solution (may be suitable for enrichment of clear beverages)	[6]
	Homogenization (VD2-βLg-low methoxyl pectin complex)	0.05% (*w*/*w*) βLg solution 0–0.15% (*w/w*) low-methoxyl pectin solution 276 μL (5 mg/mL VD2 solution) per 100 mL protein solution	PS (nm): 49–88 ζ-Potential (mV): <−40 mV	NE	The lowest turbidity (0.035) was obtained at pH 4.25 and 0.05% pectin. The optimal system was transparent and suitable for enrichment of clear acid beverages. β-Lg-pectin nanocomplex provided higher protection against VD2 degradation and stability compared to the unprotected vitamin dispersion	[49]
	Sonication and spray drying (VD3–gum arabic–chitosan complex)	9:1 (*w*/*w*) linseed oil/VD3 16% (*w*/*w*) gum arabic and chitosan as 9:1 (*w/w*) 1.5% (*w/w*) Tween 80	PS (μm): 12.64 ± 1.14 EE%: 89.78 ± 3.88	Pear juice 2 mg VD3/100 mL	Stability in quality parameters (antioxidant, physico-chemical and microbiological) after 7 days of storage at 4 °C	[44]
	Homogenization and freeze-drying (VD3–gum arabic complex)	5.0% (*w*/*v*) gum arabic solution 5 mL VD3 at concentrations corresponding to 0.3, 0.6, 3.0 and 6.0% mass of gum arabic	PS (nm): 81.3 LC: 3.47% EE%: 61.24 ζ-Potential (mV): −3.1 to −31.0 mV (pH 2.0 to 7.4)	NE	Stability at pH 2.0–7.4 range (100 days at 3 °C) High bioaccessibility (95.76%) compared to nonencapsulated VD3 (68.98%) Increased 25(OH)D levels (>81 ng/mL) after 2-week supplementation (Sprague–Dawley rats) of 60 μg VD3/day	[62]
	Homogenization (VD3–αLa–oleate complex)	VD3 (280 μM) was mixed with 4 mg/mL of αLa-oleate complex	Complete solubilization of VD3, increase in stability under UV light 9-fold, and increase in long-term stability at 37 °C up to 1000-fold	NR	The liprotide was water soluble, transparent, and protected VD3 against elevated temperatures and UV light, but was not stable at ≤pH 6 -The liprotide was suitable for enrichment of clear beverages	[63]

Not evaluated (NE); not reported (NR). Legend: α-lactalbumin (αLa); β-lactoglobulin (βLg); caprylic-/capric triglyceride (CCGT); encapsulation efficiency (EE); ethanol (EHO); glyceryl monostearate (GMS); glyceryl monostearate (GMS); loading capacity (LC); medium-chain triglyceride (MCT); methanol (MeOH); oil-in-water (O/W); particle size (PS); polyglycerol polyricinoleate (PGPR); reassembled sodium caseinate-vitamin D2 complex (RNaCas-VD); reassembled succinylated sodium caseinate-vitamin D2 complex (RSNaCas-VD), sodium caseinate complex (NaCas-VD); succinylated sodium caseinate complex (SNaCas-VD); titanium dioxide (TiO_2_); vitamin D2 (VD2); vitamin D3 (VD3); whey protein isolate (WPI); zeta potential (ζ-Potential).

**Table 3:** Vitamin D-fortified beverages currently practiced worldwide. Adapted from [7,80].

Food (Serving)	USA	Canada	Finland	Australia
	Vitamin D per Serving in μg (1 μg = 40 IU)			
Fluid cow’s milk (250 mL or 1 cup)	2.5–5.0 ^a^	2.5–5.0 ^a^	2.5–5.0 ^a^	1.25 ^b^
Orange juice with added calcium ^b^ (125 mL or 1/2 cup)	1.25	1.25	1.25	-
Plant-based milk (soy, oat, almond) ^b^ (250 mL or 1 cup)	1.5–3.0	1.5–3.0	1.9–3.75	-
Malted drink ^b^ (g powder)	3.08	-	-	-

^a^ Mandatory fortification; ^b^ Fortification of selected brands.

## Data Availability

Not applicable.
